# ”You Never Know What Happens Next” – Young Adult Service Users’ Experience with Mental Health Care and Treatment through One Year

**DOI:** 10.5334/ijic.2435

**Published:** 2016-07-22

**Authors:** Marian Ådnanes, Sissel Steihaug

**Affiliations:** 1 Research Manager, SINTEF Technology and Society, Department of Health Research, Postboks 4760, N-7465 Trondheim, Norway; 2 Senior Researcher, SINTEF Technology and Society, Department of Health Research, P.O.Box 124, Blindern, N-0314 Oslo, Norway

**Keywords:** mental health services, young adults, user experiences, treatment and care, continuity of care, Norway

## Abstract

Fragmented services are a well-known problem in the mental health sector. Mental health service users’ experiences of treatment and care can provide knowledge for developing more user-oriented continuity of care. We followed nine young adults with mental health illnesses and complex needs, conducting four interviews with each informant in the course of a year. The aim was to capture their experiences and views about treatment and care, focusing on (dis)continuities and episodes occurring through that year. The users’ experiences were affected by shifts and transitions between institutions, units and practitioners while their need was predictability and stability. A good and stable patient-provider relationship was considered highly useful but difficult to establish. The participants had a strong desire for explanation, adequate treatment and progress, but very different perceptions of the usefulness of diagnoses. Some felt rejected when they tried to tell the therapist about their trauma. Lack of user-involvement characterized many of the participants’ stories while they desired to become more engaged and included in important decisions concerning treatment and medication.

The participants’ experiences stand in contrast to key policy goals of coherent mental health services. The article discusses what may explain the gap between policy and reality, and how continuity of care may be improved.

## Introduction

The closure of hospital beds in psychiatric specialist services and the development of a more community-based mental health care system have led to greater complexity of services and made it more challenging to ensure continuity of care. Fragmented services are a well-known problem. In Norway the healthcare system is divided into two separate governmental levels: the specialist and the primary care systems, provided in accordance with different laws, regulations and funding [1]. The number of hospital beds within specialist mental health services was reduced from 48 to 10 beds per 10000 inhabitants (over 18 years old) in the period 1970–2013 [2]. In their place, district psychiatric centres have been built up under the management of specialist care. This development, together with a significant strengthening of mental health services within primary care, was carried out through a 10-year Escalation Plan conducted from 1998 to 2008 [3] resulting in the emergence of new services, disciplines, and professions, many from new interdisciplinary postgraduate studies in mental health [4,5]. The Coordination Reform was introduced in Norway in 2012 [6] to transfer tasks from the specialist level to primary care within municipalities and to improve cooperation between the levels. The patients’ need for coherent and coordinated care is considered one of the three main challenges for the Coordination Reform. Considerable emphasis is placed on developing coordinated services.

Continuity of care has become a central concept of quality in health services internationally and is also a central goal in the national Coordination Reform. It has been done several reviews in order to clarify the concept. In a recent review, Puntis et al [7] explain continuity of care as a multidimensional construct broadly defined as the long-term delivery of care that is coordinated among services and is appropriate to a patient’s current needs. Haggerty and colleagues’ [8] three-dimensional definition of relationship, informational and management continuity is a widely used example of a multidimensional approach to the concept of continuity of care. Their perspective is that the unit of measurement of continuity is fundamentally at the individual level; how patients experience integration of services and coordination. In a systematic appraisal of the literature, supplemented by interviews with patients and families, Joyce et al [9] found that continuity of care has been defined in terms of service delivery, accessibility, relationship base and individualized care. In Parker et al.’s [10] review of continuity of care-studies based on users’ experiences, they identify the following factors: users’ relationships with the providers, understanding of their condition and treatment, coordination of care, what happened to them during transition, their personal agency, and their existence as a “whole person.” Many studies show the importance of a good cooperative relationship between user and their provider [11,12,13,14].

Continuity of care in the mental health service users’ pathway has both an organizational rationale; the orderly, uninterrupted movement of patients, and an experiential aspect; health care events experienced as coherent, connected, and consistent with the user’s healthcare needs and personal context. In a recent study, we explored and identified obstacles in the movement and progress of users in relation to the organizational rational of the mental health system: lack of access to services, lack of services’ integration and inadequate coordination tools [15]. In the present study we explore the experiential considerations of continuity of care. We aim to capture how young adult mental health service users experience treatment and care, focusing on the meanings associated with (dis)continuities and episodes occurring in their pathway, in the course of one year. More knowledge based on users’ experiences may illuminate and provide a basis for developing a more user oriented continuity of care within mental health services for this group.

## Methods

Semi-structured interviews, 32 in total, were conducted quarterly with nine service users from 18 to 30 years old over the course of one year.

### Sample

Written invitations to users of mental health services were directed via clinicians in three district psychiatric centres and one hospital department, and via two municipal mental health service departments and one user organisation. No participants were recruited from the specialist services. Three participants were recruited via primary care mental health services in two different municipalities, and one via a user organisation. The remaining five participants were recruited from a secondary school for young adults with mental health problems. Eight women and one man participated.

Five of the informants told us they were diagnosed with severe mental illness; four with personality disorder and one with manic depression. Three of those without a severe mental illness had been diagnosed with depression, and one had not yet been diagnosed. Four of the nine had post-traumatic stress disorder, and three had an eating disorder. All of them mentioned anxiety as a more or less severe periodic problem. Three presently had major problems with alcohol, tablet and/or substance abuse, three had repeated problems with self-harm and eight had either once or several times taken an overdose or otherwise attempted suicidal. While two informants were relatively new receivers of mental health services, seven had long histories; 8–16 years.

Eight of the informants received work assessment allowance, one in combination with disability insurance and another in combination with a part-time job. Seven took courses at secondary school, trying to qualify for higher studies, and one studied at college. One had finished her nursing education, but started pursuing a new education at the end of the data collection.

### Data collection

We chose a qualitative interview method of data collection because interviews are suited for exploring people’s perspectives on their health care experiences [16]. In the study, attention was focused on the patients’ experience of present treatment and care and on interruption of care. The interviews were semi-structured. An interview guide was developed and used for all interviews, but the interviewer retained flexibility to adjust questions in accordance with participants’ responses. This allowed participants to fully elaborate on different or new themes. The interviews were carried out by the first author, audio-recorded and transcribed. We chose to interview participants four times during one year to closely follow their descriptions of changes during the year, and to avoid retrospective data-collection. A total of 32 interviews were conducted (two participants dropped out after one and three interviews, respectively).

The participants could choose where they wanted to be interviewed: at home, in a public place or at the researcher’s office. Most of the interviews were conducted in the interviewer’s workplace; one participant was interviewed in her care provider’s office and another was interviewed during an in-patient stay in an institution. Each interview lasted 30 to 90 minutes.

### Data analysis

The interview transcripts constitute our data. The data was analysed using a four-step analysis method of systematic text condensation suitable for analysing the material transversely and condensing information from various individuals [17]. First, we read all of the material to obtain an overall impression, focusing on the participants’ experiences with the services related to continuity and disruption. While re-reading the material, we noted preliminary topics we were able to identify in the texts. Second, we identified and classified meaningful units relevant to the aim of this study: the service users’ experience of continuity and disruption in their services. The units were developed, refined, and systematised into codes, and these codes were then assembled into code groups under appropriate headings. Third, we analysed and condensed the contents of each code group. Fourth, we summarised the condensed text in all of the code groups into an analytical text that constituted our results. Quotations from the interviews were selected to illustrate our points. In practice, our analysis was not a linear process as described here; we alternated between the various steps throughout the entire period of analysis.

### Ethics

Ethical approval for the study was obtained from the Regional Committee for Medical and Health Research Ethics (ref.: 2010/1144). Informed consent was obtained from the participants prior to the interviews. Principles of confidentiality and anonymity were applied for data collection and data analysis. Reporting and storage of data from this study is in accordance with the Act on Processing of Personal Information and the requirements of the Regional Ethics committee, based on the Health Research Act.

### Strengths and limitations of the study

The participants in our study represent a selected sample of young people, most of them attending educational programmes, many in transition to adulthood, experiencing variability and, to some extent, chaos in their lives. Their desire for change and development may be stronger than for older service users, due to being in a generally less stable phase of life and also due to a shorter period of mental health issues. Finally, eight women and one man participated, which suggests that our study does not adequately consider men’s experiences.

Despite the fact that the nine participants told different stories and struggled with various problems, they experienced many similarities and emphasised several of the same aspects of treatment. This suggests that the interviews have provided a complementary and coherent picture of users’ experiences and the meanings they attach to them.

Data analysis was carried out systematically by two experienced researchers, increasing the probability of trustworthy results. The fact that the participants were interviewed four times over the course of one year should yield more reliable results than from retrospective interviews. The interviewer compared data from multiple interviews conducted with each participant and in the last interview posed clarifying questions relating to earlier interviews. The participants could describe (/described) changes and developments in their care experiences rather than providing mere snapshots of their situation.

## Findings

The young adult service-users’ reflections on how they experienced treatment and care from the mental health services through one year can be divided into the following main issues: the problem of shifts and transitions; the important relationship between user and provider; the desire for explanation, adequate treatment, and progress; the need for information and user involvement.

### The problem of shifts and transitions

The service user’s experience of treatment and care were much affected by shifts between different institutions, units, or between practitioners, and difficult transitions between them. The participants described situations in which they had to deal with multiple practitioners because they were transferred from one unit to another or because providers left or went on leave, or because of the organization of the health service itself. Several participants also considered too many points of contact as a problem. As one participant explained:

“It does not work for me to have contact with many (providers). You have to somehow train new people each time, and it is very tiring after so many years. And it takes a very long time. I need help there and then. Then it doesn’t work with people who don’t know me and stuff, to see when I’m in a bad condition.”

The participants with additional significant substance abuse problems experienced extreme discontinuity of care because drug problems and mental health issues were treated by separate sections of specialist health care (and not simultaneously by the same provider). They desired a treatment program that would take a holistic approach to their various diagnoses and difficulties. One participant’s statement about her experience illustrates this issue:

“I miss a combined treatment. Drug abuse is at most only a symptom of something else. They haven’t realized that probably 99 percent of those with drug problems are also suffering from psychological problems in one way or another. So, if you attend substance abuse treatment, you take away the symptom, but all the reasons why people take drugs, are still there. No places (institutions/treatment programs) are suitable; either they relate to the psychological problems or they relate to the substance abuse problems. It is frustrating.”

Many participants described shortcomings with respect to cooperation between their therapists and other healthcare professionals involved in their case. Most had an individual care plan, but few felt that this worked well.

Some participants had been forced to change therapists or main providers (contact person) many times, often because the healthcare providers changed their workplace. A common reaction was then to withhold thoughts and information in the dialogue with the provider.

“I started with a psychologist in January, but this was disrupted because she became pregnant. Then I got a new one on Monday, but she is also pregnant, so she will leave in December. I have not been notified of a new one. It’s a bit difficult, because I feel I have to start over again each time I get a new therapist. It is hard to open up when you know the person is going to quit. You hold back a bit.”

Participants talked about how they needed predictability and stability; how it takes time to build trust and this requires you to have the same provider over time. The three participants that had long and established relationships with their therapist strongly emphasized the importance of this for their development. Characteristically, this applied to users whose problems were less complex in the sense that they did not include substance abuse.

### The important relationship between user and provider

The participants spoke about the importance of being “seen” as a person, being understood, respected and taken seriously, and feeling that the provider cared about them; in short, that the inter-personal “chemistry” was right:

“The chemistry works with the psychologist I have now. I felt that I was understood. She took my anxiety seriously and explained to me why it was like that. So it is probably about the person herself.”

Several participants pointed out that in order to be able to say and do the right things, the provider must also know the client. In addition, trust was important to enable the client to talk openly about difficult things. Also, directness from the therapist was also very much appreciated by some.

Many emphasised the importance of being treated with respect by their provider: not only by the single health professional but also by the institution as such. One participant recounted a stay in a specialist mental health ward where she felt she had not been treated with respect:

“The second night I suffered from a lot of anxiety, I did not dare close the door. I got out of bed again and told the night nurse that I could not sleep. I was terrified and quite shaken, but was told that I had to go to bed. (…) All I wanted was to sit in the living room with the light on, and I asked if they could be kind enough to let me sit there for 15 minutes and just read a magazine and then try to go to bed, but no, it was not allowed. They were just sending me straight to bed. So I felt like I was not a human being.”

This particular institution did not have a good reputation amongst participants. One said:

“They tend to give you up somehow”.

A participant with experience from this and other institutions told about her ongoing treatment in a small institution specialised in trauma, with a somewhat different approach to their service-users:

“The usual psychiatric services have a lot to learn from this institution, I have to say! The way they work and the way they are like people and what they stand for: their respect for individuals! I have experienced little of this in psychiatric services elsewhere.”

### The desire for explanation, adequate treatment, and progress

Participants were concerned with the cause of their mental health difficulties, but reacted differently to their diagnoses. One considered the diagnosis good to know:

“I’m going to have these challenges and I have to learn how to deal with it as best as possible.”

Another viewed her diagnosis of bipolar disorder as helpful, especially because it meant that her difficulty was “something organic” out of her control. Yet another was glad to be diagnosed with a personality disorder because this diagnosis provided an explanation for her difficulties and offered hope for her future. To her, this diagnosis meant that she was not sick, but rather that she had challenges that she could learn to cope with and tackle better through therapy:

“And then there was the way that she (her psychiatrist) convinced me; I got sort of an explanation. When she said that I was not sick, she didn’t mean that I wasn’t struggling, rather that it is not a disease. I don’t feel like I have a disease, but I have some things I need to consider. (…) Some problems are going to stay there, but then at least I have an explanation.”

Another participant disagreed with her diagnosis and perceived it to cause problems in that her thoughts and behaviour were interpreted in light of this diagnosis and providers did not really listen to what she said:

“On unit x, on the contrary, I was not understood, because they are 100 percent certain that I have a borderline personality disorder. They took this as a starting point in their treatment; what I said had no importance, and I wasn’t believed either.”

Some of the participants diagnosed with depression were critical of their diagnosis and stressed that depression was a symptom of their problems but that the diagnosis failed to address the underlying causes. Several spoke of how their diagnoses deprived them of hope:

“I was self-harming for quite a few years, and when I was 17–18 I had two overdoses in two weeks. I didn’t really want to die; I just didn’t want to feel like I was feeling. But it was because I was told that “you have a depression” and that it’s “always going to be like that.” This is incredibly stupid to say to someone of that age!”

Several were concerned with receiving appropriate treatment that would result in tangible progress, and noted that failing to make any sort of progress for a long time could cause a sense of hopelessness:

“For me it has always been important to progress. I can’t stand still with the disease I have. If I do, I loose somehow. In a case-conference I said a little discreetly that I needed progress, and then I received help to find a therapist.”

Five of the nine participants spoke explicitly of earlier trauma, for example referring to “difficult childhood.” Many pointed out that dealing with their past was their main problem, but felt rejected when raising this with their provider. One said:

“If I try to talk about something that is hard or difficult, it’s just like they try to direct my attention away from it. I wish that my therapist could go through things that are difficult (together) with me, wish he could give me some attention when I bring up stuff like that. It seems trivial to talk about the weather when I have a hard time.…”

Another said that she slowly began to talk about her history of abuse in therapy during in-patient care, but got the impression that this was not something one should talk about during treatment. As a consequence, she thought the treatment dealt only with symptoms, not their root causes. She thought it was strange, but got no explanation with respect to how the treatment was structured and no follow-up after treatment. Most participants felt that when embarking on treatment schemes, these should be at specialist level, preferably with a psychologist or psychiatrist, whilst primary care services could assist in other, more practical ways.

### The need for information and user involvement

Most participants used a passive language when they talked about how they “were admitted” and “were discharged.” Several said they were admitted to hospital without requesting in-patient stay and were then transferred from one department to another without being consulted, before ultimately being discharged before they felt ready to be so. Many described the negative effects of this uncertainty and unpredictability resulting from not being involved. One said:

“You never know what happens next; I do not know what happens this summer, I do not know what happens next fall. Nothing! I only know that the providers I now have will quit their job. I do not know if I should continue treatment in department x or not.”

Some participants felt that they did not really understand the purpose of their therapist’s approach but worried about losing the services should they express objections or fail to follow their therapist’s instructions. Others felt they were heard and had influence. One participant with a history of conflictual relationships with many different providers said that the fact that her present therapist let her participate in important decisions had been crucial in establishing a good and long-lasting relationship:

“She (her psychiatrist) made it clear that I could go at any time if I did not like her or felt that we did not work well together. (…) She had no claim on me and I did not feel trapped. I knew it was based on my own free will, and that made me choose to be there, because I had nothing to lose. She has told me that she will not be there forever, but I’ll get to decide when I want to quit.”

Some said they had specific agreements, such as short-term hospitalization when needed or scheduled admissions at regular intervals. This predictability made participants feel safe. Some also spoke about their counselling therapy and observed that they could largely control the therapy in terms of deciding which topics to discuss.

Many participants were sceptical about taking medicines and expressed a wish to participate in decisions about medication. Some had received medication without any explicit agreement on control or follow up. Some had taken overdoses on the prescribed medicines. Several used medications whose purpose they did not understand, and several thought they used too much medicine and chose to regulate the use of tablets themselves without telling their therapist. One had avoided taking medications for years, despite feeling almost forced to do so by her providers:

“They didn’t have any solutions for me. The only thing they kept saying was: “you have to start medication!” I finally got the impression that if I didn’t take medication, I didn’t deserve any help.”

Other participants felt it was ok to take medication when there was a compelling “biological reason”. One said that she had never wanted to take drugs, but she changed her mind when she was diagnosed with bipolar disorder, a diagnosis which she perceived as having a biological cause.

## Discussion

### The problem of shifts and transitions

Most of the participants experienced the services as fragmented with shifts between practitioners as well as problematic transitions between organizational boundaries. Consequently, their various problems were not seen in context, but rather treated separately by different units and practitioners.

Patients’ experience of discontinuity caused by shifts between different practitioners/clinicians is a problem referred to in many studies. Waibel et al’s [18] meta-synthesis of qualitative studies analyzing patients’ perceptions of continuity show that patients emphasize consistency of personnel when seen over time. Haggerty et al’s [19] meta-summary of patients’ experience when seeing multiple clinicians, reveals that patients find retelling their story repeatedly for new providers particularly disturbing and burdensome, while having a single trusted clinician who helps navigate the system and sees the patient as a partner provides necessary continuity. In a study of patients’ accounts of their experiences of the mental health care system, transition between key workers is found to be a source of stress and vulnerability [20].

Some changes are caused by individual choices that are difficult to prevent, such as staff leaving or changing jobs. Also the complexity of the patient’s difficulties may affect the duration of the relation. Kessler et al [21] found that persons with severe mental illnesses withdraw from contact with service providers for periods. From the provider’s point of view, working with some of these users may be perceived as challenging, and may also be affected by stigma [22]. Mental health service users may, on the other hand, obviously find shift of therapist helpful if the relationship is weak, as we have found examples of in our study. Another aspect of long-term patient-provider relations is the risk of symptoms being taken for granted (overfamiliarity) compared to being attended by a physician on a regular basis [18].

In terms of discontinuity caused by transitions between different organizations and units, the participants’ experiences confirm previous user studies. Transitions involving crossing an organizational boundary are potential breaking points, and according to Haggerty et al [19] professionals tend to forget that “every transition is a new experience for patients”. They suggest that “transition support” is needed, and that one can learn from hospital discharge planning in this respect. Another strategy to counteract these kinds of organizational breaks might be to consider other ways of organizing the services. The troubling transitions across organizational boundaries we found in our study were first and foremost between different specialised units. As a result, our participants with various diagnoses and difficulties were treated separately in different units instead of simultaneously by the same provider. This may be recognised as a break in the “consistency of care”, one of two dimensions that Haggerty et al [8] refer to as part of Management continuity (the other two are Relational and Informational continuity of care), referring to consistent and coherent management by different providers through coordinated and timely delivery of complementary services [18,19].

### The important relationship between user and provider

The participants in our study emphasised the importance of being seen and cared for during treatment, as well as being listened to, understood, and taken seriously by their provider. This related principally to their therapist, but also to the institution as such. Some recounted stories of difficult situations or periods when this had not been the case. A good patient-provider relationship, according to the participants, required “good chemistry”, trust and respect.

According to Saulz [23], patients in general strive for an interpersonal relationship that fosters trust and mutual respect. Many studies demonstrate that continuity of care is premised on a good and stable relationship between patient and provider, and that a good patient-provider relation is vital for the mental health service users to feel they are being helped [10,11,12,13,14,18,19]. Two of three studies of patients’ experience of continuity referred to the importance of the therapeutic relationship [19]. Having a single trusted clinician was considered particularly important for sensitive aspects of care, as well as for managing the patients’ multiple diagnoses [19].

Long-term relational continuity of care allowed close, collaborative relationships to develop when “fit” with clinicians was considered to be good by participants [11]. Such relationships were found to foster good illness and medication management, patient-directed decisions and aided recovery. In our study, long-term, collaborative relationships with the clinician were present among the participants whose problems were somewhat less complex, for example when not also involving substance abuse. Stable relationships between patient and provider appeared challenging to establish for the other participants. The obvious reason was the continued shifts and transitions within the system for persons with several diagnoses and/or very complex problems, including substance abuse. At the same time this group is in greatest need of stability and for the transfer of information among different units and levels of care where they receive help and care.

### Desire for explanation, adequate treatment and progress

The participants sought explanation, adequate treatment and progress, but had very different perceptions of the meaning and value of diagnosis. While some thought it was useful, others did not think the diagnosis helped them understand why they had a mental health problem and also felt the provider could then form a superficial or wrong impression of their problems.

The participants’ different views on the meaning and function of their diagnosis might reflect the fact that “diagnosis” has ambiguous meanings in today’s society. As a medical specialty, psychiatry is based on a logic in which mental disorders are classified into stable, universal categories associated with specific treatment methods with predicted effects. Many argue that a broader perspective is necessary [24,25], that the person cannot be understood separate from his or her social environment. Priebe et al [26] argue that a dominant neurobiological paradigm has resulted in a diagnostic system and approach without the individual’s experiences in their biographical and social context. Furthermore, that the dominant paradigm’s rules for research may have “stifled creativity”, and that a social paradigm could generate real progress in terms of better treatment. Psychiatry’s movement toward pure disease management, rather than a rehabilitation or healing model, might be counterproductive according to Green et al [11]. They found that patients valued competent, caring, and trustworthy clinicians who treated clinical encounters “like friendships” and supported “normal” rather than “mentally ill” identities.

Several participants in our study expressed a clear idea of their core mental health problems as well as the underlying causes, largely in the form of childhood or early youth traumas. Despite widespread trauma experience, however, several participants noted that the provider did not focus on this during treatment. Their wish of being seen as “a whole person” [10] was not met. It is well documented that there is a relationship between stressful childhood events and subsequent mental health problems [27]. This relationship is even more dramatic for mental disorders combined with substance abuse [28,29,30]. Addressing traumatisation early in the patient’s pathway seems to be an important aspect of good treatment [31,32,33]. Why were the therapists averse to delving into these young adults’ trauma experiences? The literature supports the finding that ordinary mental health services marginalize the role of trauma [34,35]. In accordance with international studies, a national study reveal that trauma or post-traumatic stress reactions are rarely mentioned as the reason for referral of children and adolescents [36]. As a result, systematic identification of traumatic experiences as part of regular intake routine in outpatient clinics is recommended by the authors. Most young adults in our study had been referred to mental health treatment as children or adolescents. Their trauma experiences and subsequent symptoms might have been ignored from the very start, thus never been an issue in treatment. Another aspect is the existing disagreement in the field as well as lack of knowledge and training in terms of how useful exposure to memories of trauma is, and in many cases, also whether it is advisable. Criteria for when it is right to help patients to confront trauma is recommended [37].

### Lack of information and user involvement

Many of the participants in our study felt they were only consulted to a limited extent, did not feel they were given sufficient information, and had little influence on the choices made for them. In general, they requested more information about what would happen next: further treatment and diagnoses, as well as medicine and medication. While many called for trauma treatment, medication was a substantial part of their treatment. However, several were sceptical about taking medication; some had felt pressured to do so and reduced the doses themselves without telling anyone.

The mental health service users’ desire for more information and participation is consistent with other studies [12,38,39]. Patients want to be part of the information loop around their care, both giving and receiving information [19]. When appropriate and timely information is absent, patients may identify transitions between services as a source of stress and vulnerability [20]. Several participants felt uncertainty and unpredictability as a result of not being involved, hence, identified with a rather passive patient-role while their desire was to be more informed and involved. When being included in important decisions, this was considered crucial to establishing a good and long-lasting relationship. Patients’ desire to be empowered to participate in decision making and have their contribution to care facilitated and recognized by the provider is well documented [18]. Giving patients choice seems to increase their engagement with services [40]. It may also be an important source for patients to overcome barriers and feel more empowered to identify solutions and establish control over their lives and illness experience [41]. It is widely recognized that mental health service users, especially those with concurrent mental health problems and substance abuse, feel disempowered, stigmatized, and disrespected, among other factors, because of the lack of influence they exert over their own situation and treatment [42,43,44].

Waibel et al [18] show that patient involvement and participation are critical factors that enhance patients’ perception of continuity of care. This presupposes the user’s participation in framing his or her treatment. However, the lack of clarity in the conceptualisation and operationalization of continuity of care has been linked to a deficit of user involvement [45].

### The gap between policy and reality

The participants’ stories about fragmented care and lack of user involvement in important decisions about treatment and medication stands in contrast to key policy goals within mental health services. What may explain the gap between policy and reality? And based on our participants’ experiences, how can continuity of care be improved?

Deinstitutionalisation and restructuring of services has resulted in a comprehensive range of mental health services, a more differentiated knowledge base and greater complexity [46]. Parallel with deinstitutionalisation, health care has become increasingly specialized, with patients seen by an increasing number of clinicians, teams, and organizations. Rapid developments in medicine’s vast knowledge have led to fragmentation of the discipline into different specialties; including within the mental health field, which now has specialties and departments for substance abuse treatment, treatment for eating disorders and so forth. This practice is not in line with health policy objectives of offering comprehensive and coherent services. It is also inconsistent with many users’ need to be seen as a ‘whole person’ [10]. An obvious strategy in order to counteract system breaks and maintain consistency in care is to avoid the splitting into separate units. Another and more refined strategy could be to exploit the power of the patient-provider relation into more than merely individual therapy. Our study exemplifies the advantage of the therapist taking a more holistic role in helping the patient to navigate the system. This role is basically due to the therapists’ particular interest and engagement in their patient, but could be developed into a formal responsibility.

Another problem is that while the evidence base for mental health services within primary care in Norway has evolved to be broader than traditional psychiatry, a schism between the different service levels seems to be present [46]. This relates to the development of two levels of knowledge, skills, and professional composition based on different knowledge bases and different organization and management principles – a development that does not contribute to integrated care. Similar to other western countries [47], despite Norway’s efforts to integrate mental health care into primary care, there seems to be a lack of clear theoretical or practical frameworks to guide such a process [46,48]. Another aspect is psychiatry’s legitimacy and development within society, expressed as skepticism towards diagnoses and medicine by our participants. Bouras [49] claims that in order to develop psychiatry’s “contract with society”, psychiatrists have, apart from being competent in clinical assessment and treatment, to pay increased attention to values as expressed by ideologies. Furthermore, that they have to work in a collaborative way with other mental health professionals as well as involve service users.

Patients’ involvement or participation is critical for continuity of care [18], and Freeman has pointed to the lack of clarity in the conceptualization and operationalization of continuity of care to a deficit of user involvement [45]. In Norway, user involvement is a statutory right where providers are urged to draw on the patient’s experience and knowledge to provide the best possible treatment and care. Still, user involvement at departmental level is deficient [50], and an agreed definition of user involvement is needed [38,39]. Based on investigating both patients’ and health personnel’s perceptions, a common definition has been identified founded on respect, dialogue and shared decision making [38], very much the same qualities sought by the participants in our study. Hence, one would expect that practicing user involvement based on these criteria would increase the chance that the users experience continuity of care.

An Individual plan for each patient is another measure and statutory right that is meant to ensure continuity of care and user participation; however, it is yet another example of an unfulfilled policy area within the field of mental health. Most of our informants had an Individual plan; however, this did not prevent fragmented care pathways [15]. National studies point to a mismatch between the rational-instrumental logic that the Individual plan stems from, and the sector’s complexity [51] combined with the fact that the services are not flexible enough to adapt to the individual’s changing needs and development [52].

### Concluding remarks

The participants’ experience of fragmented care and lack of user involvement in important decisions stands in contrast to key policy goals and years of mental health services reform. This discrepancy might reflect that knowledge and understanding within the field, as well as the organisation of services, are still dominated by a relatively traditional psychiatric disease model. Giving more attention to the epistemological problems in the mental health field and to the inequality in power and influence between the mental health care providers and between patient and provider, might contribute to better framework conditions for communication and cooperation between the different actors.
